# Pregnancy Outcomes and Documentation Status Among Latina Women: A Systematic Review

**DOI:** 10.1089/heq.2019.0126

**Published:** 2020-05-11

**Authors:** Dawn M. Richardson, Sarah B. Andrea, Amber Ziring, Cassandra Robinson, Lynne C. Messer

**Affiliations:** ^1^ Oregon Health & Science University–Portland State University School of Public Health, Portland, Oregon, USA.; ^2^ Department of Epidemiology, University of Washington School of Public Health, Seattle, Washington, USA.

**Keywords:** documentation status, immigration, pregnancy outcomes, Latina paradox, systematic review

## Abstract

**Purpose:** The impression that Latinas experience paradoxically good pregnancy outcomes in the United States persists, despite evidence showing that these outcomes are not enjoyed by all Latina subgroups. We conducted this systematic literature review to examine the relationship between documentation status and pregnancy outcomes among Latinas.

**Methods:** This review synthesizes empirical evidence on this relationship; examines how these studies define and operationalize documentation status; and makes recommendations of how a more comprehensive methodological approach can guide public health research on the impact of documentation status on Latina immigrants to the United States. We searched the literature within PubMed, Web of Science, Academic Search Premier, and Google Scholar in 2017 for relevant studies.

**Results:** Based on stringent inclusion criteria, we retained nine studies for analysis.

**Conclusion:** We found that evidence for the impact of documentation status on pregnancy outcomes among Latinas is not conclusive. We believe the divergence in our findings is, in part, due to variation in: conceptualization of how documentation status impacts pregnancy outcomes, sample populations, definitions of exposures and outcomes, and contextual factors included in models. Specific analytic challenges around sampling, measurement, and data analysis are identified. Suggestions for future research are offered regarding measurement of documentation status. Findings highlight the need for increased attention to documentation as an influence on Latina pregnancy outcomes.

## Introduction

Compared with other racial and ethnic groups in the United States, Latinas* have less education, lower socioeconomic status, less access to medical care, and lower use of prenatal care^1–3^; despite these risk factors, Latinas in the United States have surprisingly favorable pregnancy outcomes. This well-known phenomenon is the Latina paradox,^4–6^ and there is substantial evidence to support its existence and impact.^7,8^ Of critical note, with more time spent living in the United States, these paradoxically good pregnancy outcomes decline, and Latina health status draws closer to and sometimes below that of non-Latina Whites.^9–12^ Further highlighting the inequities associated with this phenomenon, the paradox has not been demonstrated or sufficiently explored across all pregnancy outcomes or stratified by documentation status. Due to the paradox, an erroneous perception has persisted that among women of color, Latina birth outcomes are not a pressing concern. Because of this prevailing view that all U.S.-based Latinas are experiencing above-optimal pregnancy outcomes (when this may not be the case), it is important to examine the paradox for variation across diverse outcomes and subgroups. Clarifying where, for whom, when, and how the paradox applies has critical implications for health equity.

Most research on the paradox has focused on low birthweight (LBW) and infant mortality (IM), finding that compared with infants of non-Latina White women, Latina infants are less likely to experience LBW^13,14^ and IM.^8^ But these are not the only outcomes of importance for Latinas and their offspring. Preclampsia, which places women at increased risk of maternal and fetal death^15^ and has implications for adverse vascular health across the life course,^16^ is more likely among Latinas than non-Latina white women^17^; similarly, Latinas—again compared with non-Latina white women—are at greater risk of hypertension,^17^ which means, among other health risks, increased risk of chronic kidney disease later in life.^18^ Further, Latinas are more likely to develop gestational diabetes mellitus (GDM), a pregnancy outcome associated with pre-pregnancy obesity^19^ and a risk factor for developing type II diabetes.^20^ In fact, half of all Latina women begin pregnancy while being either overweight or obese and experience inappropriate weight gain—both inadequate and excessive^19^—making gestational weight gain (GWG) another pregnancy outcome with nonparadoxical patterns and health implications across the life course. With the exception of women who entered pregnancy underweight,^21^ Latina women are more likely to report excessive GWG when compared with both Black and non-Black non-Latina women.^22^ This high burden of GDM and excessive GWG among Latina women places them at increased risk of giving birth to large for gestational age (LGA) infants.^23^ However, despite the Latina paradox focus on birthweight, measures of birthweight that incorporate gestational age—such as LGA and small for gestational age (SGA)—are not typically considered.

It is also notable that the paradox is not borne out across all Latina subgroups. The paradox appears to have a differential impact by nativity, with Mexican-born women experiencing better outcomes than, for example, Central or South American women.^4,8,24–27^ It is also most strongly observed among foreign-born Latinas, despite their risk profile, including higher rates of poverty and lower levels of education.^14,28^ Given the importance of nativity and nationality, a consideration of documentation status is warranted based on its impacts on immigrant well-being^29–32^; upward mobility^29,30,32^; and access to health care coverage^33–35^ and utilization.^36^

Immigration itself is a social determinant of health, and the social, political, and economic drivers of immigration and contexts of reception result in stratification with critical impacts on immigrant health across the lifecourse.^37^ Latino immigrants have encountered an increasingly hostile context of reception^38^ marked by structurally racist documentation barriers^38^ and anti-immigration policies, potentially amplifying the impact of documentation status on Latina pregnancy outcomes. Community-level factors, including social networks and social support,^39,40^ have also been pointed to as critical for Latina pregnancy health; this emphasis on social connection posits that these relationships among first-generation Latinas and the loss of these ties among second-generation Latinas (and beyond) explain the diminished pregnancy outcomes across time in the United States. These findings add to an emerging literature attempting to differentiate first- from second-generation Latina experiences. One study in this area showed that Latina immigrants experience isolation and “othering” as a result of structural and personally mediated racism^41^; another demonstrated the adverse impacts of neighborhood-level poverty and ethnic density on social processes among second-generation Latinas.^42^ At the individual level, acculturation and assimilation^43^ processes have pointed to how immigration behaviors perceived to be culturally related may shift with years of residence in the United States (possibly associated with documentation status). Immigration stress^44^ has also been pointed to as a determinant of Latina pregnancy outcomes, with stress closely linked with adverse birth outcomes. And finally, for individual Latinas, documentation status could result in differential access to health-promoting resources, since being undocumented is a known barrier for Latina immigrants in accessing prenatal care.^45–47^

In is notable that documentation status remains relatively unexplored in the research on maternal child health inequities. There are a number of reasons why research on the impacts of documentation status is limited. Concerns about a “chilling effect” among participants, manifested as reluctance to participate or fear-based dishonesty about status, have resulted in persistent hesitance by researchers to collect this information in survey-based research.^48–51^ The recognition that these data are sensitive and that gathering them have implications for harm^49,52^ has also contributed in the following manner: Collecting, storing, and analyzing documentation status (and disseminating the results) in the context of current U.S. immigrant enforcement policies places potential research participants at great risk of discovery, detention, and deportation.^53^ This barrier to scientific inquiry on the role of documentation status underscores the critical need for research aimed at understanding the relationships between being undocumented and maternal/child health. Given (1) the dearth of published research on the impact of documentation status on pregnancy outcomes; (2) our current knowledge about the inequities in outcomes across Latina subgroups; and (3) the increasingly hostile context of reception encountered by Latina immigrants to the United States, it is vital to understand the differential impacts across Latinas, specifically in documented versus undocumented women, to best meet the needs of diverse subgroups.

This systematic literature review aims to contribute to the literature by attempting to enhance our understanding of the Latina paradox by critically examining the current empirical evidence to explore how documentation status is measured and may be theorized to impact pregnancy outcomes among this population. We hypothesize that documentation status will impact pregnancy outcomes such that legal status (among foreign-born Latinas) will be protective for pregnancy outcomes (and being undocumented will increase risk for adverse outcomes). We specify this among foreign-born Latinas, because we know that U.S.-born Latinas (despite having legal status) are more likely to have worse pregnancy outcomes. This examination will further elucidate how Latinas' vulnerability to adverse outcomes is shaped and reified by documentation status. To achieve our aim, this review has three objectives: to (1) synthesize the empirical evidence on the relationship between documentation status and pregnancy outcomes among Latina women in the United States; (2) examine how these studies define and operationalize documentation status in this context; and (3) make recommendations of how a more comprehensive methodological approach can guide public health research on the impact of documentation status on Latina immigrants to the United States

## Methods

We conducted literature searches within PubMed, Web of Science, Academic Search Premier, and Google Scholar for studies that examined the association between documentation status and pregnancy outcomes (Appendix Table A1). We applied search terms (including word-form variants) systematically across all databases to capture: (1) population of interest (Hispanic, Latina); (2) exposure of interest (documentation or legal status); and (3) outcomes of interest (e.g., preterm birth [PTB], LBW, pregnancy-induced hypertension, GWG). We searched the following terms: population of interest (latin* OR hispanic* OR mexic*); exposure of interest (“immigration status” OR “legal status” OR “naturalized citizen” OR “illegal status” OR “illegals” OR “alien*” OR “undocumented” OR “documentation status” OR documented immigra* OR undocumented immigra* OR legal immigra* OR illegal immigra*); and outcomes of interest (“pregnancy weight gain” OR “pregnancy-induced hypertension” OR “pregnancy induced hypertension” OR birth outcome* OR “pregnancy outcome*” OR “eclampsia” OR “pre-eclampsia” OR “pregnancy weight” OR “postpartum” OR “low birth weight” OR “low birth-weight” OR “low birthweight” OR “small for gestational age” OR “preterm birth” OR “pre-term birth” OR “diabetes” OR “glucose” OR “gestation”). Our search was conducted in August 2017 with a subsequent manual review of reference lists.

We included English language published studies, white papers, reports, dissertations, and other literature detailing original observational research conducted in the United States. Studies were included if they: (1) included and/or restricted their study sample to Latina women; (2) quantitatively examined associations between documentation status and pregnancy outcomes; and (3) focused on Latina women from non-U.S. territories (due to our specific interest in the measurement and impact of documentation status).

### Study selection and data extraction

**FIG. 1.**
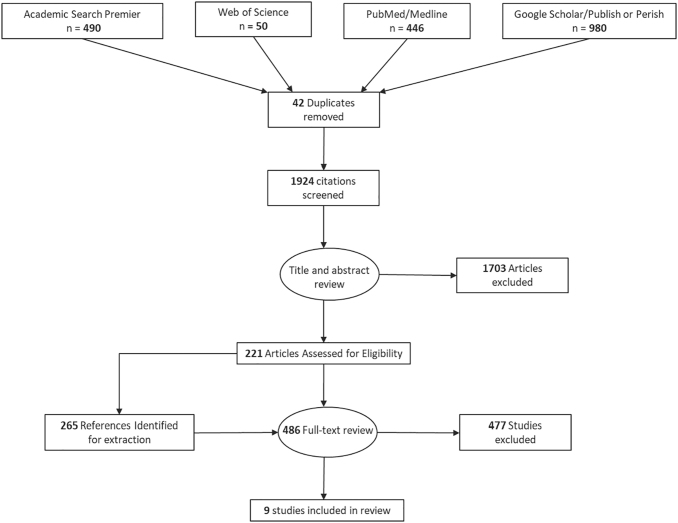
Data extraction chart.

As shown in Figure 1, the search process yielded an initial set of 1924 unique articles. Of this initial article set, 1444 were excluded based on title and abstract review, leaving 480 articles for full text review. Of those, six articles met our inclusion criteria. A review of these articles' reference lists yielded three additional articles, bringing the total for inclusion to nine.

Each paper identified in our search was independently examined by two authors. Paper titles were reviewed and excluded if they were clearly outside the review topic. If the title did not provide sufficient information to determine inclusion status, the abstract and subsequently the full text were reviewed. In the case of discrepant reviews, a third author examined the paper to determine inclusion/exclusion. Finally, this same process was applied to our review of the reference lists of the included papers.

Each author independently extracted information pertaining to the study design and analysis. To guide our review, we used the PRISMA reporting checklist, adapted as a Qualtrics abstraction form to facilitate capturing characteristics from each article, including: documentation status measurement; pregnancy outcomes definition and ascertainment; race/ethnicity and country of origin of study sample; covariates; and statistical approach, including management of missing data. To assess each included study's resiliency from bias, we used a modified version of the NIH Quality Assessment Tool for Observational Cohort and Cross-sectional Studies (Appendix A1), with two authors independently appraising each study. Given that one purpose of this review is to report the quality of research in this area and make recommendations for future research, we include all studies in this review—irrespective of resiliency from bias—as is consistent with the emerging nature of this research topic.

This study was exempted by the Portland State University institutional review board.

## Results

Of the 1924 articles initially identified through our review process, only 6 met our full inclusion criteria; another 3 articles found through reference checks brought the total reviewed articles to 9. Exclusion of abstracts was primarily due to study populations not inclusive of Latinas and/or not capturing pregnancy outcomes. Exclusion of full-text articles resulted when the study did not specify and measure documentation status and/or pregnancy outcomes were limited to adequacy of prenatal care.

Studies examined nine unique cohorts of women and their infants born from 1980 through 2008, utilizing birth records, claims data, and/or in-person interviews to procure data. For all but two studies,^54,55^ outcomes were ascertained via administrative data or medical record extraction. These two studies captured outcome measures directly from participants: The first^54^ ascertained information about cesarean deliveries via self-report, and the second^55^ assessed postpartum depression with the Center for Epidemiologic Studies Depression scale. The majority of studies were restricted to Latina women with variable nativity; in four studies,^26,54,56,57^ Mexico was the country of origin for all or most of the women; and one additional study included a substantial percentage of Mexican-origin Latinas.^55^ The nine reviewed studies examined U.S. populations sourced from seven states; of these, two used data from three states traditionally receiving immigrants: Texas, California, and New York in one study^58^ and California, New York, and Florida in another.^55^ Four total studies utilized data from California,^54–56,58^ three from New York,^47,55,58^ two from Texas,^58,59^ and one each for Utah,^26^ Massachusetts,^60^ Colorado,^57^ and Florida^55^ (Table 1).

**Table 1.** Study Characteristics in the Reviewed Studies

First author, year	Study design	Data source	Location	Time period	Race/ethnicity	Country of origin	Undocumented determination
Chavez, 1986	Cross-sectional	Snowball population-based sample recruited for in-home interviews	San Diego, CA	1981–1982	100% Latina	100% Mexico	Self-report
Geltman, 1999	Cross-sectional	Women consecutively approached in postpartum hospital wards	Boston, MA	Not disclosed	Not disclosed	54% United States 19% Haiti 6% Caribbean 5% Central America 4% Cape Verde 2% Puerto Rico	Self-report
Kalofonos, 1999	Cross-sectional	Health records and interviews with women who had given birth at the UCSD Medical Center	San Diego, CA	1997–1998	100% Latina	85% Mexico 15% United States	No social security number and/or self-report
Joyce, 2001	Ecological time-series	Birth Records	California New York City, NY Texas	1989–1998	100% Latina	United States Mexico Dominican Republic Other Latin American Countries (Proportions not disclosed)	Foreign-born and uninsured
Kelaher, 2002	Geographically defined retrospective cohort	MIC-Women's Health Services Clinical Records	New York City, NY	1997–1997	76% Latina 24% Latina and Black	31% Dominican Republic 26% United States 14% Mexico 9% Ecuador 5% El Salvador 4% Columbia 3% Honduras 3% Guatemala 5% Other South and Central American Countries	No social security number or resident status card at intake
Kuo, 2004	Cross-sectional	Women consecutively approached in postpartum hospital wards	Brooklyn, NY San Francisco, CA Miami, FL	1999–2001	100% Hispanic	57.7% Cuba 35.9% Mexico 26.1% Central America 13.4% Dominican Republic 10.8% South America [13% U.S.-born]	Self-report
Reed, 2005	Geographically defined retrospective cohort	Birth records linked to Medicaid claims	Colorado	1998–1999	Not disclosed	93% Mexico^a^	Emergency Medicaid usage
Dang, 2011	Geographically defined retrospective cohort	CHIP Perinatal and Medicaid claims	Texas Gulf Coast Region	2008	43.9% Hispanic^b^ 31.1% White non-Hispanic 23.3% Black non-Hispanic 1.5% Asian 0.3% American Indian 0.1% Other	Not disclosed	CHIP Prenatal Insurance
Flores, 2012	Geographically defined retrospective cohort	Birth records	Utah	2004–2007	84% White 16% Latina	81% Mexico^c^	No social security number

^a^ Among emergency Medicaid users. Country of origin for Medicaid users (both U.S.-born and presumably foreign-born documented not disclosed).

^b^ Race/ethnicity data only available for Medicaid claims. However, authors conducted a surname analysis and concluded and “overwhelming majority” of CHIP Prenatal are Hispanic.

^c^ Among foreign-born Latinas (12.5% of study population).

CHIP, Children's Health Insurance Program.

Documentation status was determined based on self-report in three studies^54,55,60^; the remaining studies relied on absence of social security number^26,47,56^ and/or emergency Medicaid usage^57–59^ as proxies for undocumented status. Notably, two of the three studies employing emergency Medicaid status as a proxy for undocumented status did not disclose the race/ethnicity^57^ and/or country of origin.^59^ Seven studies^26,47,56–60^ examined the impact of documentation status on birth outcomes. Each of these studies considered continuous birthweight (or dichotomized LBW), making it the most frequently assessed outcome. Three of the seven studies^26,57,59^ examining birth outcomes found that undocumented status was associated with lower odds of dichotomized PTB and/or LBW infants (Table 2). Two of these studies^26,57^ specified this outcome among Mexican origin women; the remaining study^59^ did not specify nativity but was based in Texas. In contrast, the authors of one study^26^ observed that undocumented foreign-born Latinas had greater odds of giving birth to SGA infants than documented foreign-born Latinas before adjustment for maternal factors, including pregnancy complications; notably, this study yielded mixed results and also found no statistically significant associations with LBW and protective impacts on PTB. An additional study's^60^ examination of continuous birthweight yielded a gradient whereby, on average, infants born to documented foreign-born mothers were the largest and infants born to U.S.-born mothers were the smallest.

**Table 2.** Summary of Studies Examining Undocumented Status as a Predictor of Adverse Pregnancy and/or Birth Outcomes

First author, year	Population comparison	Outcomes measured	Undocumented outcome association	Strengths	Limitations
Birth outcomes					
Dang, 2011	TX CHIP Perinatal users with unknown race/ethnicity and country of origin compared with all TX Medicaid users	LBW^a^	**↓**	• Large sample size • Inclusive of a vulnerable population	• Inadequate covariate adjustment; race/ethnicity of population using CHIP Prenatal unknown • Emergency Medicaid imperfect proxy for documentation status • Complete case analyses with known differential missingness of outcome data (31% for CHIP prenatal, 10% for Medicaid)
		PTB^b^	**↓**		
Flores, 2012	UT foreign-born Latinas without SSNs compared with foreign-born Latinas with SSNs; 81% of Mexican origin	LBW^a^	◦	• Includes relevant covariates • Utilized a census of Utah births • Appropriate ascertainment of documentation status	• Covariate selection strategy not well justified; adjusted for factors that may be mechanisms through which documentation status affects health
		PTB^b^	**↓**		
		SGA^c^	**↑**^d^		
Geltman, 1999	MA self-reported documented and undocumented foreign-born women from a variety of countries (predominantly Haiti) compared with U.S.-born women	Birthweight (g)	U.S.-born < undocumented foreign-born < documented foreign-born	• Explicit measure of documentation status • Short time period between outcome occurrence and data collection.	• Selection bias (consecutive sampling; women not interviewed when interpreter unavailable) • Time period not disclosed • Inadequate covariate adjustment; race/ethnicity unknown • Linear examination of continuous birthweight
		Gestational age (weeks)	U.S.-born < undocumented foreign-born and documented foreign-born		
Joyce, 2001	CA, TX, and NY foreign-born and U.S.-born insured and uninsured Latinas before and after PRWORA	Change in LBW^a^ post-PRWORA	**↑**^e^	• Strong pre**–**post policy design • Extensive covariate adjustment • Census of all births in study locations during study periods, with the exception of those with missing data	• Weak proxy for undocumented status • Some of the covariates (e.g., smoking illicit drugs) poorly represented on vital records; may introduce more bias than they correct for
Kalofonos, 1999	CA foreign-born Latinas without SSNs compared with foreign-born and U.S.-born Latinas with SSNs; all Mexican	LBW^a^	◦	• Mixed methods; included medically under-served • Variables abstracted from medical records (not self-report) • Explicit measure of documentation status available for some	• Small sample size • Selection bias: LBW estimates are based on a sample in which all women with limited prenatal care were included but only a random sample of women with adequate prenatal care • Some covariates included in adjusted model may be mechanisms through which documentation status affects health
Kelaher, 2002	NY foreign-born Latinas without SSNs or residency cards compared with U.S.-born Latinas; predominantly Dominican Republic country of origin	LBW^a^	◦	• Proxy measure for documentation status developed/employed in prior research • Large sample size • Country of origin considered (but not in relation to documentation status)	• Previous low-birth-weight birth outcome may introduce sample selection • Possible misclassification of documentation status (by use of proxy measure) • Data come from prenatal service data source, therefore women not receiving prenatal care are not represented in research
Birth and pregnancy outcomes					
Reed, 2005	CO Emergency Medicaid users of predominantly Mexican origin compared with Medicaid users of unknown race/ethnicity	LBW^a^	**↓**	• Considered wide range of pregnancy outcomes • Included behavioral mediators of pregnancy outcomes (smoking, drinking) • Statewide cohort of undocumented women	• Emergency Medicaid as imperfect proxy for documentation status • 14% of claims files that did not match a birth record or matched multiple records • Results generalizable to singletons • Complete case analyses
		PTB^b^	**↓**		
		Cesarean delivery	◦		
		Complications of delivery^f^	**↑**		
		Abnormal conditions of newborn^g^	**↑**		
Pregnancy outcomes					
Chavez, 1986	CA self-reported undocumented compared with documented foreign-born women; all of Mexican origin	Cesarean delivery	**↓**	• Recruitment tactics optimized to achieve representative sample of undocumented people • Well-defined measure of documentation status • In-depth interviews resulted in both quantitative and qualitative data	• Potential selection bias: sample dependent on snowball sampling “seed” or initial interview • Small sample size • Unadjusted proportions and chi-square tests presented; no adjustment for potential confounding
Kuo, 2004	NY, CA, and FL self-reported undocumented compared with documented foreign-born Hispanic women; predominantly Cuban and Mexican origin	Postpartum depression^h^	↑^d^	• Explicit measure of documentation status developed in consultation with legal professionals • Considered nonbirth pregnancy outcome • Large sample size	• Descriptive statistics suggest differences across recruitment sites; however, analyses do not account for clustering by site • Some covariates included in the adjusted model may be mechanisms through which documentation status affects health • CESD depression definition (cut points) not adjusted for factors that may be a function of having recently delivered a live birth)

◦ No association; Significant negative ↓ or positive ↑ association.

^a^ <2500 g with the exception of Kalofonos (<3000 g).

^b^ <37 Weeks.

^c^ <10th Percentile of birthweight for gestational age and sex.

^d^ Significant before adjustment.

^e^ Only significant for NYC Other Latinas.

^f^ Includes meconium staining, excessive bleeding, premature rupture, precipitous labor, malpresentation, cord prolapse, and fetal distress.

^g^ Includes infant anemia, birth injury, fetal alcohol syndrome, hyaline membrane disease, seizures, and requirements for assisted ventilation.

^h^ CES-D ≥ 16.

CHIP, Children's Health Insurance Program; CI, confidence interval; GWG, gestational weight gain; LBW, low birthweight; OR, odds ratio; PRWORA, Personal Responsibility and Work Opportunity Reconciliation Act; PTB, preterm birth; SGA, small for gestational age.

Three studies^54,55,57^ examined the relationship between documentation status and pregnancy outcomes. In minimally adjusted models, one^57^ found that undocumented status was associated with higher odds of pregnancy complications and another^55^ found that undocumented status was associated with postpartum depression. Adequate covariate adjustment was defined by our study team as adjustment for: maternal age, education, and marital status and was observed in none of the included studies. Two studies^54,60^ included no covariates, one study^59^ adjusted for maternal age only, and three studies^26,55,56^ adjusted for factors that are potential consequences of documentation status as covariates (e.g., employment status, health insurance status, pregnancy complications). Three studies^26,57,59^ excluded multiple births and very preterm and/or LBW births; two studies^54,55^ excluded births to women younger than the age of 18.

The nine studies overall met more than 60% of the quality parameters, with missing data being the most frequent study quality issue in this review. Notably, the proportion of the study sample with missing observations (and sociodemographic characteristics of those with missing observations) was seldom reported—all studies performed complete case analyses. In studies that did report on missingness, differential missingness was observed (e.g., 31% Children's Health Insurance Program [CHIP] prenatal vs. 10% Medicaid missing^59^). The number of quality parameters met by each study can be found in Appendix Tables A2 and A3, and the implications of unmet quality parameters are examined in the discussion.

## Discussion

Based on our systematic review, evidence for the impact of documentation status on pregnancy outcomes among Latinas is not conclusive. Our hypothesis—that among foreign-born Latinas documented status would prove protective for pregnancy outcomes—was not wholly borne out, with our finding of divergent associations across outcomes. Undocumented status was generally either not associated or associated with lower odds of PTB and LBW; however, we also saw that being undocumented was associated with greater odds of pregnancy complications, abnormal conditions of the newborn, and postpartum depression. Given the heterogeneity of the studies (with regard to populations included, variable definitions of exposures and outcomes, and the diversity of contextual factors considered), the inconsistency was unsurprising. Until researchers engage in more standardized approaches, the true effects of documentation status on pregnancy outcomes may remain unclear.

One influence on our findings could be the different causal pathways leading to each unique outcome; elucidating these pathways has important implications for advancing health equity. For example, stress, which we hypothesized to be differentially experienced by documentation status and is a known risk factor for PTB and LBW, was found to be protective for these outcomes. This may be because this pathway is not as sensitive to immigration stress as expected, or that this stress is experienced too proximally to the pregnancy outcome to be adverse. Or it could be that all Latina women, whether documented or not, may be experiencing stress resulting from fear for family members or friends who may be undocumented and identified as such, or from having their own documentation status questioned. Therefore, the literature, as it stands, may be unable to distinguish the physiological stress resulting from documentation status from the chronic stress experienced by the Latina community overall. Given the evidence on social support among Latinas, it may also be that these strong relationships are, in fact, mitigating immigrant stress in ways that limit its adverse impacts, despite evidence that these ties are challenging for immigrants to maintain. Or it could be that the benefits associated with being foreign born are so strong that any impacts resulting from lack of documentation are not sufficiently adverse to neutralize them. We did expect to find that the protective effects conferred by foreign-born status would be diminished when compounded by undocumented status, with the lack of legal documentation “overriding” the protective effects resulting in the Latina paradox, and here our results and expectations aligned.

For those outcomes in our sample of studies not associated with physiological stress (e.g., pregnancy complications, postpartum depression, or unintended cesarean section), undocumented status was found to be associated with greater odds of occurrence. This set of outcomes is more directly related to poor patient**–**provider communication, inadequate prenatal care, or nonadherence to clinical recommendations and could, therefore, be more sensitive to documentation (and not protected by social ties), with undocumented status potentially leading to increased discomfort with or inability to communicate with medical care providers regarding pain experienced, birthing preferences, or other emerging issues. Future research examining the role of documentation status should ensure to consider the immigrant social ties hypothesis as well as patient**–**provider interactions to tease out these relationships.^61^

In addition to these explanations, which focus on how documentation status is conceptualized to impact pregnancy outcomes, we identify multiple analytical issues that may have limited our ability to see clear relationships in the reviewed literature.

### Challenge 1: population inclusivity

The most vulnerable undocumented women may not be properly represented in the reviewed literature. For studies conducting interviews with Latina women, differential representation may be based on challenges specific to the research questions (e.g., fears over revealing documentation status, linguistic barriers). In addition, in the absence of an interpreter, undocumented women may be systematically excluded for being disproportionately non-English speaking; one reviewed study^60^ highlights this reliance on Spanish-language interpreter availability as an ultimate influence for participants. Further, undocumented Latinas may be underrepresented by virtue of where recruitment occurs, and even when included may be excluded from final models due to differential missingness.^59^

### Challenge 2: measurement of documentation status

Revealed in this set of literature is a reliance on proxy measures for ascertaining documentation status. Our inconsistent findings may be due in part to this use of proxies since they provide an indirect assessment of the complex relationship between documentation status and pregnancy outcomes. For example, two studies^57,59^ employed emergency Medicaid utilization as an indicator of undocumented status; however, this is apparently the closest approximation available, and it is merely a proxy for legal status. Three additional studies categorized participants as undocumented if they could not produce a social security number^26,56^ or resident status card^47^ at intake. Again, although a reasonable approach, this method does not guarantee specificity in measurement of documentation status and could result in mis-categorization^49^ and dilution of the potential effect of documentation status.

### Challenge 3: heterogeneity of sampled population

Another potential cause of inconsistencies across the findings could stem from populations sampled: Of the nine reviewed studies, only five included predominantly Mexican foreign-born samples. The other studies included a range of Latina subgroups, which is important for our understanding of the findings because the Latina paradox is most robust among Mexican-born women and immigrant experience varies by nativity.^62^ An example of this sampling issue is found in one study,^47^ which included a sample that was only 14% Mexican-born, finding that documentation status was not associated with LBW, a result that may be due to the authors' use of an all foreign-born comparison group, an analytic decision that could have resulted in a “washing away” of the effect of paradox. Across the reviewed literature, undocumented women were frequently compared with women of heterogeneous nativity status: Only three of the studies compared undocumented foreign-born Latinas with a group consisting solely of documented foreign-born Latinas or only U.S.-born Latinas. Because we hypothesized the poorest outcomes among our U.S.-born Latinas and the best outcomes among our documented foreign-born Latinas, null results could be explained by an even mixture of the two and potentially seemingly “protective” findings could be resulting from sampled populations where there are more U.S.-born than foreign-born Latinas.

### Challenge 4: adjustment/model specification

A number of studies included variables in their regression models that could actually be in the causal pathway between documentation status and pregnancy outcome. Specific covariates varying based on documentation status include: presence/number of prenatal care visits, insurance status, employment status, and substance use^56^; smoking^58^; income, employment, and health insurance coverage^55^; and prepregnancy BMI, smoking, and alcohol use.^26^ These factors are hypothesized to explain part of the relationship between documentation status and the observed outcomes, so this over-adjustment could bias results toward the null. In contrast, others^54,59,60^ incorporate minimal or no covariate adjustment in their analyses and/or do not restrict evaluation of birth outcomes to singleton births.

Given mixed findings on the impact of documentation status on pregnancy outcomes across the reviewed literature, a full understanding of the relationship between this exposure and the outcomes of interest remains elusive. The outcomes reported here suggest that attempts to elucidate this relationship would be enhanced by clear theorizing on the pathways leading to impact; analytic strategies that reflect these conceptualizations; consistent measurement of documentation status and pregnancy outcomes; and sampling appropriate to investigating these relationships. We recommend that researchers clarify the specific ways in which they believe that being undocumented would impact pregnancy outcomes, which will guide the selection of appropriate outcomes to be examined.^50^ In addition, we would hope to see this conceptual work reflected in the sample selection, which—if done in ways that recognize barriers to participation (e.g., legal status, language barriers)—would bolster findings and aid understanding of any identified associations.^50^

Our final recommendation pertains to the measurement of documentation status: Researchers should limit the use of proxy measures when feasible. As previously discussed, the collection of legal status data carries tremendous risk for research participants, and public health researchers have been vocal about the need for caution in collecting this information.^50,52^ The majority of our reviewed studies were published at least 20 years ago, and the climate around immigration and documentation status has shifted, leading toward an increasingly precarious position for undocumented research participants. Fortunately, guidance exists on how to engage in the collection of such data in ethical, scientifically responsible ways that can advance our understanding of documentation status impacts on health. Specific datasets (e.g., LA FANs) have incorporated precautions to protect undocumented participants and are considered well suited for such examinations.^48,51^ A recent review of documentation status measurement in health research suggested a move away from proxy measures to self-report,^49^ building on prior recommendations to combine survey and ethnographic approaches.^63^ Others^50^ offer specific strategies for protecting participants while ensuring data validity, and addressing issues related to navigating IRB applications and securing informed consent, providing^51^ a model for how to engage in this work in culturally responsive ways. Ultimately, legal protections and controls are warranted when asking for this information.^52^

Our review has some important limitations. First, our search was limited to articles that were written in English, which may introduce language bias. However, because the focus of our study was the United States, we believe that there are less likely to be missing papers written in other languages. Second, although we performed a robust review including Google Scholar—which is useful for finding gray literature—our review may be subject to publication bias. Third, because of the heterogeneity in outcomes, exposure definitions, and groups being compared, we were unable to conduct a formal meta-analysis. Fourth, despite including several key words for pregnancy outcomes that occur disproportionately among Latina women, such as GWG, gestational diabetes, and LGA, none of the eligible studies examined these outcomes. Finally, the small sample size coupled with afore mentioned heterogeneity reduced our ability to make strong inferences about the relationship between documentation status and pregnancy and birth outcomes in Latina women.

To our knowledge, this work constitutes the first systematic review of the impacts of documentation status on Latina pregnancy outcomes. Our findings highlight the need for further examination of the role of legal status by showing that being undocumented in the United States can adversely impact the health of women and their offspring, with far-reaching potential for health and health inequities across the life course. Researchers engaging in this work should consider the challenges we describe here—related to theory, sampling, measurement, and modeling—and consider the related recommendations when developing studies to examine documentation status and pregnancy outcomes among Latinas.

## Abbreviations Used

CHIP: Children's Health Insurance Program
CI: confidence interval
GDM: gestational diabetes mellitus
GWG: gestational weight gain
IM: infant mortality
LBW: low birthweight
LGA: large for gestational age
OR: odds ratio
PRWORA: Personal Responsibility and Work Opportunity Reconciliation Act
PTB: preterm birth
SGA: small for gestational age

## Appendix

### Appendix A1. Study extraction form and modified NIH Quality Assessment Tool for Observational Cohort and Cross-Sectional Studies

**Paper Extraction: Documentation Status and Pregnancy/Birth Outcomes—September 2017**

Q16 Date (mm/dd/yyyy)
___________________________________________________________________________________
Q10 Reviewer
○ Amber (1)
○ Cassandra (2)
○ Dawn (6)
○ Lynne (3)
○ Sarah (4)
○ Stella (5)

Q1 Paper ID # (must be 3–4 digits)
___________________________________________________________________________________

Q13 First Author Last name
___________________________________________________________________________________

Q14 Year of Study
___________________________________________________________________________________
Q19 ***Study Characteristics***
Q51 What Type of Study Design?
□ Ecological (1)
□ Cross-Sectional (2)
□ Prospective Cohort (6)
□ Geographically Defined Retrospective Cohort Study (vital records) (3)
□ Mixed Methods (qual/quant) (4) ________________________________________________
□ Other (5) ________________________________________________
Q9 Aims or Objectives of the Study (What was the intention as reported in the paper?)
___________________________________________________________________________________
___________________________________________________________________________________
___________________________________________________________________________________
___________________________________________________________________________________
___________________________________________________________________________________
Q20 Inclusion Criteria (as written in the report):
___________________________________________________________________________________
___________________________________________________________________________________
___________________________________________________________________________________
___________________________________________________________________________________
___________________________________________________________________________________
Q22 Exclusion Criteria (as indicated in the report):
___________________________________________________________________________________
___________________________________________________________________________________
___________________________________________________________________________________
___________________________________________________________________________________
___________________________________________________________________________________
Q34 Time Period that the Study Represents:
___________________________________________________________________________________
Q24 Recruitment Procedures Used (if not indicated, type “unknown.” If study utilized vital records, indicate “not applicable.”)
___________________________________________________________________________________
___________________________________________________________________________________
___________________________________________________________________________________
___________________________________________________________________________________
___________________________________________________________________________________
Q25 Geographic Location (city, state)
___________________________________________________________________________________
___________________________________________________________________________________
___________________________________________________________________________________
___________________________________________________________________________________
___________________________________________________________________________________
Q61 Study Context or Settings (hospital, clinic, population, multiple cities, etc.)
___________________________________________________________________________________
Q46 This is the end of the study characteristics section. Do you want to proceed to End Extraction?
○ Yes, indicate why (1) ________________________________________________
○ No (2)
Skip To: Q40 If Q46=1
Q26 ***Participant Characteristics***
Q27 Overall Size of the Study (this includes people who met inclusion criteria and not excluded)
___________________________________________________________________________________
Q47 Number of Undocumented Women Included in the Analysis (if known):
___________________________________________________________________________________
Q29 Race and/or Ethnicity (as specified in the language of the study):
___________________________________________________________________________________
___________________________________________________________________________________
___________________________________________________________________________________
___________________________________________________________________________________
___________________________________________________________________________________
Q30 Country of Origin:
___________________________________________________________________________________
___________________________________________________________________________________
___________________________________________________________________________________
___________________________________________________________________________________
___________________________________________________________________________________
Q62 Additional Identifier (e.g. surname, language spoken, refugee status, time in country, etc.)
___________________________________________________________________________________
___________________________________________________________________________________
___________________________________________________________________________________
___________________________________________________________________________________
___________________________________________________________________________________
Q58 This is the end of the participant characteristics section. Do you want to proceed to End Extraction?
○ Yes, indicate why (1) ________________________________________________
○ No (2)
Skip To: Q40 If Q58=1
Q32 ***Independent Variables***
Q33 How was Documentation Status Ascertained?
□ Implied (weird proxies, etc) (1) ________________________________________________
□ Explicit/Clear/Direct (2) ________________________________________________
□ Unclear (will discuss/review within the group) (3) ________________________________________
□ Documentation status not addressed (end extraction) (4)
Skip To: Q40 If Q33=4
Q36 How is Documentation Status Defined or Described (exact language from author)?
___________________________________________________________________________________
___________________________________________________________________________________
___________________________________________________________________________________
___________________________________________________________________________________
___________________________________________________________________________________
Q49 If paper includes time in country or time since immigration, please indicate how they addressed time-related information here:
___________________________________________________________________________________
Q35 Adjusting Covariates/Confounders (searched for/controlled for/adjusted for):
___________________________________________________________________________________
___________________________________________________________________________________
___________________________________________________________________________________
___________________________________________________________________________________
___________________________________________________________________________________
Q37 Modifying Variables/Effect Measure Modifiers (search for stratification or interaction terms):
___________________________________________________________________________________
___________________________________________________________________________________
___________________________________________________________________________________
___________________________________________________________________________________
___________________________________________________________________________________
Q60 This is the end of the independent variables section. Do you want to proceed to End Extraction?
○ Yes, indicate why (1) ________________________________________________
○ No (2)
Q38 ***Outcomes***
Q39 Select all birth and pregnancy outcomes examined in the study and define them if possible.
□ Low Birth Weight (1) ________________________________________________
□ Birth Weight (2) ________________________________________________
□ Pre-term Birth (3) ________________________________________________
□ Large for Gestational Age (4) ________________________________________________
□ Small for Gestational Age (5) ________________________________________________
□ Pregnancy-Induced Hypertension (6) ________________________________________________
□ Pre-eclampsia (7) ________________________________________________
□ Eclampsia (8) ________________________________________________
□ Gestational Weight Gain (high, low, appropriate, or continuous) (9) ________________________________________________
□ Gestational Diabetes (13) ________________________________________________
□ Abortion (10) ________________________________________________
□ Depression (specify antenatal or postnatal measurement) (11) ________________________________________________
□ Anxiety (specify antenatal or postnatal measurement) (12) ________________________________________________
□ Other (14) ________________________________________________
Q38 How were outcomes collected*? (select all that apply)*
□ Self-report (interview, survey) (1)
□ Objective measures by study personnel (2)
□ EHR/EMR (5)
□ Existing Dataset (indicate dataset) (3) ________________________________________________
□ Other (4) ________________________________________________
Q61 This is the end of the outcomes section. Do you want to proceed to End Extraction?
○ Yes, indicate why (1) ________________________________________________
○ No (2)
Skip To: Q40 If Q61=1
Q54 ***Study Results and Analysis***
Q55 What type of analysis was used? Copy/paste the analytic plan from the methods section of the paper.
___________________________________________________________________________________
Q56 Results
○ Odds Ratio (1)
○ Risk Ratio (2)
○ Beta Coefficients (3)
○ Prevalence Estimates (4)
○ Other (5) ________________________________________________
Q57 If possible, attach a screenshot of the results table here (1):
Q63 Details from Table 1:
□ Covariates adjusted for (1) ________________________________________________
□ Analytic sample (2) ________________________________________________
□ How they dealt with missing data (3) ________________________________________________
Q58 If possible, attach a screenshot of the results table here (2):
Q64 Details from Table 2
□ Covariates adjusted for (1) ________________________________________________
□ Analytic sample (2) ________________________________________________
□ How they dealt with missing data (3) ________________________________________________
Q59 If possible, attach a screenshot of the results table here (3):
Q65 Details from Table 3
□ Covariates adjusted for (1) ________________________________________________
□ Analytic sample (2) ________________________________________________
□ How they dealt with missing data (3) ________________________________________________
Q66 Please indicate any narrative results here:
___________________________________________________________________________________
___________________________________________________________________________________
___________________________________________________________________________________
___________________________________________________________________________________
___________________________________________________________________________________
Q77 What are the authors' primary conclusions? This is typically in the first paragraph in the discussion or conclusion section of the paper.
___________________________________________________________________________________
___________________________________________________________________________________
___________________________________________________________________________________
___________________________________________________________________________________
___________________________________________________________________________________
Q78 What are the primary study limitations (as identified by the author)?
___________________________________________________________________________________
___________________________________________________________________________________
___________________________________________________________________________________
___________________________________________________________________________________
___________________________________________________________________________________
Q59 This is the end of the study results section. Do you want to proceed to End Extraction?
○ Yes, indicate why (1) ________________________________________________
○ No, continue to quality review (2)
Skip To: Q40 If Q59=1
Q52 ***Quality Review***
Quality Assessment Tool for Observational Cohort and Cross-Sectional Studies
For additional guidance: https://www.nhlbi.nih.gov/health-pro/guidelines/in-develop/cardiovascular-risk-reduction/tools/cohort
Q60 1. Was the research question or objective in this paper clearly stated?
Did the authors describe their goal in conducting this research? Is it easy to understand what they were looking to find? This issue is important for any scientific paper of any type. Higher quality scientific research explicitly defines a research question.
○ Yes (1) ________________________________________________
○ No (2) ________________________________________________
○ Other (CD, cannot determine; NA, not applicable; NR, not reported) (3) ________________________________________________
Q62 2. Was the study population clearly specified and defined?
Did the authors describe the group of people from which the study participants were selected or recruited, using demographics, location, and time period? If you were to conduct this study again, would you know who to recruit, from where, and from what time period? Is the cohort population free of the outcomes of interest at the time they were recruited?
○ Yes (1) ________________________________________________
○ No (2) ________________________________________________
○ Other (CD, cannot determine; NA, not applicable; NR, not reported) (3) ________________________________________________
Q63 3. Was the participation rate of eligible people at least 50%?
If fewer than 50% of eligible people participated in the study, then there is concern that the study population does not adequately represent the target population. This increases the risk of bias.
○ Yes (1) ________________________________________________
○ No (2) ________________________________________________
○ Other (CD, cannot determine; NA, not applicable; NR, not reported) (3) ________________________________________________
Q64 4. Were all the subjects selected or recruited from the same or similar populations (including the same time period)? Were inclusion and exclusion criteria for being in the study prespecified and applied uniformly to all participants?
○ Yes (1) ________________________________________________
○ No (2) ________________________________________________
○ Other (CD, cannot determine; NA, not applicable; NR, not reported) (3) ________________________________________________
Q65 5. Was a sample size justification, power description, or variance and effect estimates provided?
A paragraph in the methods section of the article may explain the sample size needed to detect a hypothesized difference in outcomes. You may also find a discussion of power in the discussion section (such as the study had 85% power to detect a 20% increase in the rate of an outcome of interest, with a two-sided alpha of 0.05). Sometimes, estimates of variance and/or estimates of effect size are given, instead of sample size calculations. In any of these cases, the answer would be “yes.” However, observational cohort studies often do not report anything about power or sample sizes because the analyses are exploratory in nature. In this case, the answer would be “no.” This is not a “fatal flaw.” It just may indicate that attention was not paid to whether the study was sufficiently sized to answer a prespecified question—that is, it may have been an exploratory, hypothesis-generating study.
○ Yes (1) ________________________________________________
○ No (2) ________________________________________________
○ Other (CD, cannot determine; NA, not applicable; NR, not reported) (3) ________________________________________________
Q66 6. For the analyses in this paper, were the exposure(s) of interest measured before the outcome(s) being measured?
If a cohort study is conducted properly, the answer to this question should be “yes,” since the exposure status of members of the cohort was determined at the beginning of the study before the outcomes occurred. Because in retrospective cohort studies the exposure and outcomes may have already occurred (it depends on how long they follow the cohort), it is important to make sure that the exposure preceded the outcome.
Sometimes, cross-sectional studies are conducted (or cross-sectional analyses of cohort-study data), where the exposures and outcomes are measured during the same timeframe. As a result, cross-sectional analyses provide weaker evidence than regular cohort studies regarding a potential causal relationship between exposures and outcomes. For cross-sectional analyses, the answer to Question 6 should be “no.”
○ Yes (1) ________________________________________________
○ No (2) ________________________________________________
○ Other (CD, cannot determine; NA, not applicable; NR, not reported) (3) ________________________________________________
Q67 7. Was the timeframe sufficient so that one could reasonably expect to see an association between exposure and outcome if it existed?
The issue of timeframe is important to enable meaningful analysis of the relationships between exposures and outcomes to be conducted. This often requires at least several years, especially when looking at health outcomes, but it depends on the research question and outcomes being examined. Cross-sectional analyses allow no time to see an effect, since the exposures and outcomes are assessed at the same time, so those would get a “no” response.
○ Yes (1) ________________________________________________
○ No (2) ________________________________________________
○ Other (CD, cannot determine; NA, not applicable; NR, not reported) (3) ________________________________________________
Q68 8. Were the exposure measures (independent variables) clearly defined, valid, reliable, and implemented consistently across all study participants?
Were the exposure measures defined in detail? Were the tools or methods used to measure exposure accurate and reliable—for example, have they been validated or are they objective? This issue is important, as it influences confidence in the reported exposures. When exposures are measured with less accuracy or validity, it is harder to see an association between exposure and outcome even if one exists. Also as important is whether the exposures were assessed in the same manner within groups and between groups; if not, bias may result.
○ Yes (1) ________________________________________________
○ No (2) ________________________________________________
○ Other (CD, cannot determine; NA, not applicable; NR, not reported) (3) ________________________________________________
Q69 9. Were the outcome measures (dependent variables) clearly defined, valid, reliable, and implemented consistently across all study participants?
Were the outcomes defined in detail? Were the tools or methods for measuring outcomes accurate and reliable—for example, have they been validated or are they objective? This issue is important, because it influences confidence in the validity of study results. Also important is whether the outcomes were assessed in the same manner within groups and between groups.
○ Yes (1) ________________________________________________
○ No (2) ________________________________________________
○ Other (CD, cannot determine; NA, not applicable; NR, not reported) (3) ________________________________________________
Q70 10. Were the outcome assessors blinded to the exposure status of participants?
Blinding means that outcome assessors did not know whether the participant was exposed or unexposed. It is also sometimes called “masking.” The objective is to look for evidence in the article that the person(s) assessing the outcome(s) for the study (for example, examining medical records to determine the outcomes that occurred in the exposed and comparison groups) is masked to the exposure status of the participant. Sometimes, the person measuring the exposure is the same person conducting the outcome assessment. In this case, the outcome assessor would most likely not be blinded to exposure status because they also took measurements of exposures. If so, make a note of that in the comments section. As you assess this criterion, think about whether it is likely that the person(s) doing the outcome assessment would know (or be able to figure out) the exposure status of the study participants. If the answer is no, then blinding is adequate. An example of adequate blinding of the outcome assessors is to create a separate committee, whose members were not involved in the care of the patient and had no information about the study participants' exposure status. The committee would then be provided with copies of participants' medical records, which had been stripped of any potential exposure information or personally identifiable information. The committee would then review the records for prespecified outcomes according to the study protocol. If blinding was not possible, which is sometimes the case, mark “NA” and explain the potential for bias.
○ Yes (1) ________________________________________________
○ No (2) ________________________________________________
○ Other (CD, cannot determine; NA, not applicable; NR, not reported) (3) ________________________________________________
Q71 11. Was loss to follow-up after baseline 20% or less?
Higher overall follow-up rates are always better than lower follow-up rates, even though higher rates are expected in shorter studies, whereas lower overall follow-up rates are often seen in studies of longer duration. Usually, an acceptable overall follow-up rate is considered 80% or more of participants whose exposures were measured at baseline. However, this is just a general guideline. For example, a 6-month cohort study examining the relationship between dietary sodium intake and BP level may have more than 90% follow-up, but a 20-year cohort study examining effects of sodium intake on stroke may have only a 65% follow-up rate.
○ Yes (1) ________________________________________________
○ No (2) ________________________________________________
○ Other (CD, cannot determine; NA, not applicable; NR, not reported) (3) ________________________________________________
Q72 12. Were key potential confounding variables measured and adjusted statistically for their impact on the relationship between exposure(s) and outcome(s)?
Were key potential confounding variables measured and adjusted for, such as by statistical adjustment for baseline differences? Logistic regression or other regression methods are often used to account for the influence of variables that are not of interest. This is a key issue in cohort studies, because statistical analyses need to control for potential confounders, in contrast to an RCT, where the randomization process controls for potential confounders. All key factors that may be associated with both the exposure of interest and the outcome—which are not of interest to the research question—should be controlled for in the analyses.
○ Yes (1)
○ No (2)
○ Other (CD, cannot determine; NA, not applicable; NR, not reported) (3) ________________________________________________
Q75 Guidance for Overall Quality Rating
The questions just cited are designed to help you focus on the key concepts for evaluating the internal validity of a study. They are not intended to create a list that you simply tally to arrive at a summary judgment of quality.
I nternal validity for cohort studies is the extent to which the results reported in the study can truly be attributed to the exposure being evaluated and not to flaws in the design or conduct of the study—in other words, the ability of the study to draw associative conclusions about the effects of the exposures being studied on outcomes. Any such flaws can increase the risk of bias.
Critical appraisal involves considering the risk of potential for selection bias, information bias, measurement bias, or confounding (the mixture of exposures that one cannot tease out from each other). Examples of confounding include co-interventions, differences at baseline in patient characteristics, and other issues throughout the questions cited earlier. High risk of bias translates to a rating of poor quality. Low risk of bias translates to a rating of good quality. (Thus, the greater the risk of bias, the lower the quality rating of the study.)
I n addition, the more attention in the study design to issues that can help determine whether there is a causal relationship between the exposure and outcome, the higher the quality of the study. These include exposures occurring before outcomes, evaluation of a dose-response gradient, accuracy of measurement of both exposure and outcome, sufficient timeframe to see an effect, and appropriate control for confounding—all concepts reflected in the tool.
Generally, when you evaluate a study, you will not see a “fatal flaw,” but you will find some risk of bias. By focusing on the concepts underlying the questions in the quality assessment tool, you should ask yourself about the potential for bias in the study you are critically appraising. For any box where you check “no,” you should ask, “What is the potential risk of bias resulting from this flaw in study design or execution?” That is, does this factor cause you to doubt the results that are reported in the study or doubt the ability of the study to accurately assess an association between exposure and outcome?
The best approach is to think about the questions in the tool and how each one tells you something about the potential for bias in a study. The more you familiarize yourself with the key concepts, the more comfortable you will be with critical appraisal. Examples of studies rated good, fair, and poor are useful, but each study must be assessed on its own based on the details that are reported and consideration of the concepts for minimizing bias.
Q67 Final thoughts on quality review to discuss among the group:
___________________________________________________________________________________
___________________________________________________________________________________
___________________________________________________________________________________
___________________________________________________________________________________
___________________________________________________________________________________
Q40 Comments or additional information not previously indicated:
___________________________________________________________________________________
___________________________________________________________________________________
___________________________________________________________________________________
___________________________________________________________________________________

**Appendix Table A1.** Search Terms by Database

Source	Criteria		
	Population: Latina women	Exposure: documentation status	Outcome: pregnancy and/or birth outcomes
Academic Search Premier	latin^*^ OR hispanic^*^ OR mexic^*^	“immigration status” OR “legal status” OR “naturalized citizen” OR “illegal status” OR “illegals” OR “alien^*^” OR “undocumented” OR “documentation status” OR documented immigra^*^ OR undocumented immigra^*^ OR legal immigra^*^ OR illegal immigra^*^	“pregnancy weight gain” OR “pregnancy-induced hypertension” OR “pregnancy induced hypertension” OR birth outcome^*^ OR “pregnancy outcome^*^” OR “eclampsia” OR “pre-eclampsia” OR “pregnancy weight” OR “postpartum” OR “low birth weight” OR “low birth-weight” OR “low birthweight” OR “small for gestational age” OR “preterm birth” OR “pre-term birth” OR “diabetes” OR “glucose” OR “gestation”
Web Of Science	TS=(latin^*^ OR hispanic^*^ OR mexic^*^)	TS=(“immigration status” OR “legal status” OR “naturalized citizen” OR “illegal status” OR illegals OR alien^*^ OR undocumented OR “documentation status” OR documented immigra^*^ OR undocumented immigra^*^ OR legal immigra^*^ OR illegal immigra^*^)	TS=(“pregnancy weight gain” OR “pregnancy-induced hypertension” OR “pregnancy-induced hypertension” OR birth outcome^*^ OR “pregnancy outcome^*^” OR eclampsia OR pre-eclampsia OR “pregnancy weight” OR postpartum OR “low birth weight” OR “low birth-weight” OR “low birthweight” OR “small for gestational age” OR “preterm birth” OR “pre-term birth” OR diabetes OR glucose OR gestation
Pubmed/Medline	latin^*^ OR hispanic^*^ OR mexic^*^	“immigration status” OR “legal status” OR “naturalized citizen” OR “illegal status” OR “illegals” OR “alien^*^” OR “undocumented” OR “documentation status” OR documented immigra^*^ OR undocumented immigra^*^ OR legal immigra^*^ OR illegal immigra^*^	“pregnancy weight gain” OR “pregnancy-induced hypertension” OR “pregnancy induced hypertension” OR birth outcome^*^ OR “pregnancy outcome^*^” OR “eclampsia” OR “pre-eclampsia” OR “pregnancy weight” OR “postpartum” OR “low birth weight” OR “low birth-weight” OR “low birthweight” OR “small for gestational age” OR “preterm birth” OR “pre-term birth” OR “diabetes” OR “glucose” OR “gestation”
Google Scholar/Publish or Perish	latino OR latina OR hispanic OR mexican	“immigration status” OR “legal status” OR “naturalized citizen” OR “illegal status” OR “illegals” OR “alien^*^” OR “undocumented” OR “documentation status” OR documented immigra^*^ OR undocumented immigra^*^ OR legal immigra^*^ OR illegal immigra^*^	“pregnancy weight gain” OR “pregnancy-induced hypertension” OR “pregnancy induced hypertension” OR birth outcome^*^ OR “pregnancy outcome^*^” OR “eclampsia” OR “pre-eclampsia” OR “pregnancy weight” OR “postpartum” OR “low birth weight” OR “low birth-weight” OR “low birthweight” OR “small for gestational age” OR “preterm birth” OR “pre-term birth” OR “diabetes” OR “glucose” OR “gestation”

**Appendix Table A2.** Study Results: Explicit Determination of Documentation Status

First author, year	Outcomes measured	Exclusions	Covariates	N^a^	OR (95% CI)	No. of quality criteria Met^b^
Birth outcomes						
Flores, 2012	LBW^c^ PTB^d^ SGA^e^	• Multiple births • Records with missing data for ethnicity, maternal birth place, gestational age, and birthweight • Birthweight <200 g or >5499 g • Gestational age <20 weeks	• Maternal age • Parity • Maternal education • Maternal medical risk factors • Presence of pregnancy complication • Smoking and alcohol use during pregnancy • Prepregnancy BMI	196,617^f^ (13,208 Undocumented)	[Documented vs. Undocumented foreign-born Latinas] LBW unadjusted: 0.93 (0.82–1.04) LBW adjusted: 1.08 (0.95–1.24) PTB unadjusted: 1.05 (0.95–1.17) PTB adjusted: 1.15 (1.02–1.30) SGA unadjusted: 0.82 (0.75–0.91) SGA adjusted: 0.93 (0.83–1.05)	9
Geltman, 1999	Birthweight (gram) Gestational age (week)	• Women who did not complete the interview	Not applicable	171 (20 Undocumented)	[means] Birthweight (*p* < 0.01 for US vs. non-US) • U.S.-Born, 3135 • Documented, 3435 • Undocumented, 3357 Gestational age • U.S.-Born, 38.7 • Documented, 39.5 • Undocumented, 39.5	5
Kalofonos, 1999	LBW^c^	• Missing observations	• Maternal age • Parity • No. of prenatal care visits • Insurance status • Marital status • Employment status • Gravity • Substance use	173^g^ (91 Undocumented)	[Ref: U.S.-born and documented Mexican women] Self-report: 1.74 (0.56–5.41) No SSN: 0.40 (0.15–1.11)	6
Kelaher, 2002	LBW^c^	• Women without previous live births • Missing observations	• Maternal age • Maternal education • Parity • Maternal medical risk factors	4975^g^ (915 Undocumented)	[Ref: U.S.-born] Undocumented foreign-born: 0.9 (0.7–1.2) Documented foreign-born: 0.8 (0.6–0.9)	9
Pregnancy outcomes						
Chavez, 1986	Cesarean delivery	• ≤17 Years old • Residing and working outside San Diego County • Women who gave birth in the United States >5 years before interview • Missing observations	Not applicable	235 (138 Undocumented)	[Ref: Documented foreign-born] 0.39 (0.21–0.71)^h^	6
Kuo, 2004	Postpartum depression^i^	• <17 years old • No grandparent of Latin American or Latin Caribbean national or ethnic origin • Puerto Rican or Brazilian • Unable to communication in Spanish or English • Maintaining residence in study area <6 months from enrollment • Medical or psychological condition that could impede study interview • Unable to give informed consent • Missing observations	• Maternal age • Maternal education • Income • Marital status • Employment • Health insurance coverage	3952^g^ (1970 Undocumented)	[Ref: U.S.-born and documented foreign-born Latinas] Unadjusted: 1.59 (1.38–1.83) Adjusted: 0.97 (0.76–1.23)	8

^a^ No. of participants included in the analytic sample.

^b^ Modified version of the NIH Quality Assessment Tool for Observational Cohort and Cross-sectional Studies.

^c^ <2500 g with the exception of Kalofonos (<3000 g).

^d^ <37 Weeks.

^e^ <10th Percentile of birthweight for gestational age and sex.

^f^ Variable analytic sample: 3651 missing in the adjusted models.

^g^ Analytic sample as stated in the article but adjusted models are a complete case. Unclear how many women were included in adjusted models.

^h^ ORs calculated from reported proportions.

^i^ CES-D ≥ 16.

CI, confidence interval; GWG, gestational weight gain; LBW, low birthweight; OR, odds ratio; PTB, preterm birth; SGA, small for gestational age.

**Appendix Table A3.** Study Results: Implicit Determination of Documentation Status

First author, year	Outcomes measured	Exclusions	Covariates	N^a^	OR (95% CI)	No. of quality criteria Met^b^
Birth outcomes						
Dang, 2011	LBW^c^ PTB^d^	• Birthweight <500 g • Multiple births • Insurance besides CHC CHIP or Medicaid • Missing observations	• Maternal age	15,377	[Ref: CHIP perinatal] LBW 2.2 (1.9–2.6) PTB 2.1 (1.8–2.4) LBW or PTB 1.9 (1.7–2.2)	8
Joyce, 2001	Change in LBW^c^ post-PRWORA	• Puerto Rican Births • Missing observations	• Maternal age • Marital status • Maternal educational attainment • Parity • Infant sex • Year	Not disclosed	[Ref: U.S.-born] CA Mexicans 1.06 (0.99–1.12) CA Other Latinas 0.97 (0.81–1.16) NYC Dominicans 1.33 (0.94–1.87) NYC other Latinas 1.28 (1.02–1.62) TX Mexicans 1.02 (0.95–1.09) TX Other Latinas 1.00 (0.82–1.21)	9
Birth and pregnancy outcomes						
Reed, 2005	Cesarean delivery Complications of delivery^e^ Abnormal conditions of newborn^f^ LBW^c^ PTB^d^	• Claims not related to delivery • Multiple births • Missing observations	• Smoking status • Gestational weight gain • Maternal age (individually controlled for)	118,904^g^ (5961 Undocumented)	[Ref: Non-Emergency Medicaid Insurance] Cesarean delivery 0.95 (0.89–1.03)^h^ Complications 1.84 (1.74–1.94)^h^ Abnormal conditions of newborn 1.31 (1.20–1.43)^h^ LBW 0.81 (0.72–0.90)^h^ PTB 0.87 (0.81–0.94)^h^	8

^a^ No. of participants included in the analytic sample.

^b^ Modified version of the NIH Quality Assessment Tool for Observational Cohort and Cross-sectional Studies.

^c^ <2500 g.

^d^ <37 Weeks.

^e^ Includes meconium staining, excessive bleeding, premature rupture, precipitous labor, malpresentation, cord prolapse, and fetal distress.

^f^ Includes infant anemia, birth injury, fetal alcohol syndrome, hyaline membrane disease, seizures, and requirements for assisted ventilation.

^g^ Analytic sample as stated in article but adjusted models are a complete case. Unclear how many women were included in adjusted models.

^h^ ORs calculated from reported proportions.

CHIP, Children's Health Insurance Program; PRWORA, Personal Responsibility and Work Opportunity Reconciliation Act.
